# AI driven automation for enhancing sustainability efforts in CDP report analysis

**DOI:** 10.1038/s41598-025-07584-4

**Published:** 2025-07-07

**Authors:** Ramya Rangarajan, Tamilarasi Kathirvel Murugan, Logeswari Govindaraj, Venyaa Venkataraman, Krithik Shankar

**Affiliations:** School of Computer Science and Engineering, Vellore Institute of Technology, Chennai, India

**Keywords:** Sustainability, Carbon disclosure project, Genetic algorithm, Long short-term memory, Emissions optimization, Resource efficiency, Environmental impact, Atmospheric science, Climate change, Machine learning, Quality control, Electrical and electronic engineering

## Abstract

The need for sustainable practices in supply chains is becoming increasingly critical, as businesses face pressure to reduce their carbon footprint while maintaining operational efficiency. This paper proposes a novel hybrid approach that combines Genetic Algorithms (GA) with Long Short-Term Memory (LSTM) networks to optimize supply chain sustainability. The proposed system leverages publicly available Carbon Disclosure Project (CDP)-reported data to predict emissions and optimize resource allocation. The primary objective of this research is to develop a cost-effective, scalable solution that reduces emissions, improves operational efficiency, and ensures regulatory compliance within supply chains. The hybrid model consists of two main components: LSTM networks for predictive modeling of emission trends and GA for optimization of supply chain processes. LSTM is used to forecast future emissions based on historical data, while GA optimizes resource management, including transportation choices and energy consumption, to minimize emissions and operational costs. The system employs a multi-objective optimization approach, addressing the simultaneous goals of emission reduction, operational efficiency, and compliance with environmental regulations. The experimental results demonstrate the effectiveness of the proposed approach. A 23.67% reduction in total emissions was achieved, with the most significant improvements in indirect emissions. The system also improved operational efficiency by 10.98%, while ensuring 100% compliance with environmental regulations, eliminating any penalties. The hybrid GA-LSTM framework offers valuable insights for businesses seeking to meet sustainability targets and provides a practical, data-driven method for improving supply chain performance. The proposed system is not only applicable to large corporations but can also be scaled for use in small and medium-sized enterprises, offering a pathway for widespread adoption of sustainable practices across industries.

## Introduction

The growing impact of climate change underscores the urgent need for sustainability across industries. Among the most pressing concerns are carbon emissions, energy inefficiencies, and excessive resource consumption. Governments, corporations, and consumers increasingly prioritize sustainability, aiming to reduce environmental impact while ensuring operational efficiency and financial viability. In this regard, cutting-edge data-driven strategies like artificial intelligence (AI) and optimization algorithms have become effective instruments for addressing sustainability issues. The CDP provides a critical platform for organizations to report their environmental performance, including emissions data, energy usage, and other sustainability metrics. Despite its standardized datasets, many organizations underutilize CDP reports, relying on static analysis methods that fail to capture dynamic environmental challenges effectively. Additionally, real-time IoT sensor data, though useful, is often expensive, complex to deploy, and raises concerns regarding data privacy and scalability. Thus, leveraging structured CDP data offers an alternative that is both accessible and cost-efficient.

To address these challenges, this study proposes a hybrid framework integrating LSTM networks and GA to optimize sustainability outcomes. LSTM networks leverage historical data trends to forecast emissions, while GA optimizes the forecasts by balancing emissions reduction, cost efficiency, and regulatory compliance. This integration removes the dependency on expensive real-time data collection and offers a scalable sustainability solution for businesses of all sizes.

### Problem statement

The challenge of sustainability optimization lies in balancing the economic viability of businesses with the need for environmental stewardship. Traditional optimization techniques often fail to capture the dynamic interdependencies within environmental systems, necessitating the adoption of AI-driven solutions. By leveraging structured CDP data, this study introduces a predictive and optimization-based approach that minimizes environmental impact while ensuring business competitiveness. The framework enables businesses to forecast emissions using historical data trends, then apply GA to optimize multi-objective goals, including emissions reduction, cost efficiency, and compliance with regulations.

### Proposed AI-driven framework

Artificial intelligence is a game-changer when it comes to solving sustainability issues because it allows businesses to examine vast amounts of data, spot inefficiencies, and streamline processes. The proposed framework integrates two key AI techniques: LSTM networks for emissions forecasting and GA for sustainability optimization.

#### LSTM networks for emissions forecasting

LSTM networks are a type of recurrent neural network (RNN) that excels at processing sequential data and capturing long-term dependencies, making them ideal for predicting trends in emissions and resource consumption. The ability to analyze past emissions trends enables LSTMs to forecast future emissions, providing businesses with the foresight needed to plan proactive sustainability strategies. Given the structured nature of CDP data, these networks are able to effectively handle nonlinear trends and long-term relationships inherent in environmental data. As a result, LSTM-based models offer accurate predictions, empowering organizations to anticipate and mitigate emissions growth before it exceeds targeted thresholds.

#### Genetic algorithms for multi-objective optimization

The GA is an optimization technique inspired by the principles of natural selection, which evolves a population of candidate solutions to identify optimal outcomes. In the context of sustainability, GAs can help organizations navigate the complex trade-offs between conflicting objectives, such as reducing carbon emissions, minimizing operational costs, and adhering to regulatory standards. By evolving solutions over successive generations, GA ensures that the final solution optimally balances these competing priorities, yielding strategies that minimize emissions while enhancing business performance.

GA provides a crucial advantage in sustainability optimization because it can consider multiple factors simultaneously and adjust solutions dynamically, accounting for the complex, non-linear relationships present in sustainability challenges.

### Primary outcomes and contributions

This research offers a novel approach to sustainability optimization by integrating LSTM-based forecasting and GA optimization into a hybrid framework. The primary outcomes of this study include:

Accurate Emissions Forecasting: By using historical emissions data from CDP reports, the LSTM network produces precise forecasts of future emissions trends. This allows businesses to anticipate potential increases in their carbon footprint and implement corrective actions proactively.

Multi-Objective Optimization: The GA enables businesses to optimize the trade-offs between emissions reduction, cost-efficiency, and regulatory compliance. This multi-objective optimization ensures that sustainability goals are met without compromising economic performance, addressing the need for cost-effective environmental strategies.

Cost-Effective Sustainability Monitoring: Unlike conventional methods that rely on expensive real-time IoT sensors, the proposed framework utilizes structured CDP data, which is accessible, standardized, and already available to many organizations. This reduces the cost and complexity of implementing sustainability monitoring systems, making them more accessible to businesses of all sizes.

Scalable and Adaptive Framework: The hybrid framework is designed to be scalable across different industries and business sizes. It adapts to changing environmental conditions and evolving regulations, ensuring long-term sustainability improvements while maintaining business competitiveness.

The structure of the paper is organized as follows. Section II presents a comprehensive review of related work, examining existing AI-driven and optimization-based approaches to sustainability and outlining their key limitations, particularly in relation to data dependence, scalability, and predictive capability. Section III describes the proposed hybrid methodology, detailing the data acquisition process from CDP reports, the architecture of the LSTM network for emissions forecasting, and the integration of GA for multi-objective optimization. Section IV outlines the experimental setup and presents the results of the empirical evaluation, demonstrating the effectiveness of the framework in reducing emissions while maintaining operational efficiency. Section V discusses the practical implications of the findings, the broader impact on industry and policy, and the inherent limitations of the proposed approach. Finally, Section VI concludes the study and provides insights into future research directions aimed at enhancing the scalability, accuracy, and adaptability of AI-driven sustainability optimization systems.

## Related works

In an era of rapid technological advancements and increasing global interconnectivity, the need for sustainable practices has become more pressing than ever. Climate change, resource depletion, and environmental degradation are among the critical challenges facing industries worldwide. Addressing these issues requires an urgent focus on reducing carbon emissions, optimizing resource utilization, and promoting environmentally responsible operations. Central to these efforts is the integration of advanced technologies, such as AI, to analyse environmental data and develop solutions that align with sustainability objectives. The use of fossil fuels greatly increases greenhouse gas emissions, which exacerbates global warming, carbon emissions and environmental deterioration.

Organizations increasingly recognize the benefits of reducing carbon footprints, which include cost savings, regulatory compliance, and improved resilience to climate-related risks. As industries prioritize sustainable operations, frameworks such as the Technology Acceptance Model and Diffusion of Innovations are essential for understanding the barriers to adopting these practices. These frameworks help guide decision-making for businesses across various sectors by addressing environmental awareness and policy constraints^1^. Solar energy integration plays a critical role in reducing carbon footprints and addressing fossil fuel dependency, particularly in developing countries. It indicates that integrating solar energy can significantly reduce carbon footprints while addressing the challenges of fossil fuel dependency, thus promoting sustainable development in the energy sector^2^. The relevancy of energy and carbon footprint assessment in distributed and federated learning has increased exponentially especially within the realms of Industry 4.0 and 5G technologies. This solidifies the importance of sustainable artificial intelligence solutions, examining the balance between centralized and decentralized learning methods^3^.

Natural Language Processing (NLP) techniques can be used to enhance automated systems’ interpretability, particularly when calculating carbon footprints from banking transactions. Furthermore, the study of model-agnostic approaches yields good results in terms of effective performance and user confidence, addressing the lack of transparency usually associated with machine learning models in industrial settings^4^. In higher education, AI-tools for predictive analysis help access and reduce carbon footprints from operational activities, such as transportation and energy use. These tools support the transition towards more sustainable educational institutions, aligning them with global sustainability goals^5^.

The design process becomes more difficult due to the complexity of many hardware-software interactions, which results in inefficiencies and higher energy usage. Moreover, the absence of sustainability consciousness in resource allocation in current operating systems calls for the creation of optimization frameworks and algorithms that maximize resource efficiency while reducing operational emissions across a range of workloads^6^. AI and big data analytics can be used to optimize low-carbon energy economies and demonstrates important developments in raising energy efficiency and lowering carbon emissions. Big data technologies like cloud computing and the Internet of Things in conjunction with a variety of AI methods like neural networks and particle swarm optimization can be used to accomplish this^7^.

There is a need to optimize resource placement to mitigate energy consumption and CO2 emissions. Furthermore, Mixed-Integer Linear Programming (MILP) models and the integration of renewable energy resources can improve energy efficiency in data centres, are critical to the growing demand for cloud computing^8^. The Monte Carlo simulations hold great significance in assessing the environmental impact of land use and resource management. This contributes more to sustainable rural economies. By integrating ecological, sociological, and economic aspects, industries can better evaluate sustainability efforts in various sectors^10^. Emerging technologies like Big Data and AI play a major role in advancing the United Nations Sustainable Development Goals (SDGs). International standards from organizations like ISO, IEEE, and ITU promote sustainable industrialization, digital inclusion, and economic development, requiring further empirical evaluation^11^. NLP techniques analyse emotional sentiment from unstructured data, such as financial news, improving carbon price forecasting models. Advanced algorithms like MS-IHHO-LSTM enhance predictions by integrating sentiment with market data, emphasizing the role of public perception in carbon pricing^12^.

It is undoubted that evaluating carbon emissions in manufacturing and emphasizing on the importance of low-carbon manufacturing processes because of their high energy consumption and greenhouse gas emissions is vital. Optimizing sustainability efforts in energy-intensive industries, such as cement production, is also made possible through AI-driven solutions. By adjusting machining parameters in real-time, Big Data analytics help reduce carbon emissions in manufacturing^13^. Integrated strategies play a major role in fostering sustainable economic growth. Green energy generation and demand-side control measures provide essential insights into how energy and economic policies can be aligned to reduce climate change while promoting sustainable development^14^.

Technology like non-intrusive load monitoring (NILM) has proven effective in optimizing energy consumption by understanding electric vehicle (EV) charging patterns, which is crucial for managing carbon emissions. By integrating social, psychological, and economic factors, energy prediction models can become even more effective in promoting sustainable energy management^15^. AI extends beyond energy optimization into carbon footprint evaluation and accounting. Machine learning models calculate real-time emissions, helping corporations reduce their carbon footprint. These models are particularly useful in smart cities, where real-time data on emissions and energy use enhances sustainability, highlighting the importance of data-driven carbon management^16^.

Adopting sustainability practices is driven by environmental, economic, and social imperatives. Benefits include cost savings, regulatory compliance, enhanced reputation, and climate resilience. Consumer demand for transparency pushes industries to prioritize sustainability. Integrating sustainability throughout the technology lifecycle proves to reduce carbon emissions while improving efficiency. This can significantly lower carbon emissions while simultaneously improving operational efficiency^17^. Cutting-edge communication technologies, such as the Internet of Things and machine learning, are essential in enhancing smart buildings’ sustainability and energy efficiency. However, there are shortcomings in the comprehensive frameworks that integrate several technologies, suggesting that more research is necessary to address problems with data security and interoperability^18^.

Sustainability is supported by transparency-enabling technologies like blockchain and digital platforms, which help organizations track environmental data, verify compliance, and provide stakeholders with reliable information. Blockchain plays a key role in carbon accounting and emissions reporting, ensuring industries meet corporate carbon reporting rules with accountability and trust^19^. The integration of machine learning algorithms like Support Vector Regression (SVR) and Neural Networks (NN) are useful in predicting and optimizing carbon emissions. These models help develop sustainable concrete solutions, aligning with the UN’s Sustainable Development Goals. Incorporating energy-efficient production techniques in concrete manufacturing is vital for reducing emissions in the construction sector^20^.

The study^21^ proposes a blind super-resolution framework for fabric defect detection, addressing image quality and dataset limitations by using Real-ESRGAN and Poisson noise degradation. The authors introduce a novel local blur discriminative loss function, enhancing image sharpness for defect detection. Experimental results on the DAGM2007 dataset show significant improvements in accuracy, recall, and mean average precision (mAP) compared to traditional augmentation methods. In^22^, the authors tackle rate-dependent hysteresis in piezoelectric actuators by reformulating the Dahl model into a neural network structure (DahlNN), coupled with a Layer Recurrent Network (LRN). This interpretable hybrid model enhances motion control precision by reducing modeling errors by 97.3% and fluctuations by 70%, outperforming standard LRN models with minimal computational overhead. The study in^23^ introduces a Visual Question Answering (VQA) system for carbon neutrality, combining computer vision and natural language processing to interpret environmental images and provide actionable insights. The VQA framework helps identify carbon emission hotspots, assess green initiatives, and support evidence-based policy-making, promoting sustainability and climate change mitigation.

In^24^, a deep learning-based intrusion detection framework for the Internet of Vehicles (IoV) is proposed, incorporating advanced encryption techniques like AES-256 and Homomorphic Encryption (HE) to ensure privacy. The system uses wavelet transforms, Vision Transformers (ViT), and Graph Attention Networks (GAT) for feature extraction, with a hybrid DAGSNet architecture for efficient model training. Evaluations on CAN and CIC-IDS2017 datasets demonstrate exceptional performance, achieving over 98% accuracy while maintaining fast encryption speeds. The study^25^ proposes a novel compensation method for magnetic encoder errors, integrating Variational Mode Decomposition (VMD) with a Deep Belief Network (DBN) optimized using Particle Swarm Optimization (PSO). This model significantly improves the accuracy of angle detection in robotic systems, reducing angular errors from 0.22° to 0.0025° and outperforming traditional machine learning models. In^26^, machine learning techniques are applied to predict the axial load capacity of Elliptical Double Steel Columns (EDSCs), with the AdaBoost model achieving the highest accuracy (R^2^ = 0.996) and robust generalization capabilities. The study provides a user-friendly graphical interface for structural engineers to improve safety and efficiency in construction. Lastly,^27^ reviews the emerging field of intelligent auditing, emphasizing its application beyond financial contexts in public sector auditing. The study explores the challenges in automating audits using machine learning and anomaly detection and calls for interdisciplinary collaboration to create more transparent, scalable auditing systems.

The study by^28^ introduced a privacy-preserving framework for early cardiac disease detection using federated learning and attention-based feature fusion, integrating multi-modal data such as cardiac images, ECG signals, patient records, and nutrition data, achieving high accuracy (97.76%, 98.43%, 99.12%) across datasets. Similarly,^29^ developed a breast cancer classification model with Seagull Optimization Algorithm (SGA) for gene selection and Random Forest (RF) for classification, reaching 99.01% accuracy with only 22 genes, outperforming traditional models. In the neurodevelopmental domain,^30^ proposed an EEG-based ADHD detection system using NeuroDCT-ICA preprocessing and ADHD-AttentionNet, achieving 98.52% accuracy, while^31^ presented a composite framework for zero-day exploit detection in cybersecurity, combining AWPA, MATA, GMCO, and AHEDNet to achieve up to 99.2% accuracy and minimal Hamming Loss. These studies highlight the effective application of advanced AI techniques in healthcare diagnostics and cybersecurity threat detection. As shown in Table 1, the comparison of research approaches across various domains reveals distinct techniques, objectives, and innovations that align with specific goals in each field.

**Table 1:** Comparison of research approaches in diverse domains: techniques, objectives, and innovations.

Aspects	^21^	^22^	^23^	^24^	^25^	^26^	^27^	^28^	^29^	^30^	^31^
Domain	Textile defect detection	Robotics & control systems	Environmental AI	Cybersecurity (IoV)	Robotics/automation	Civil/structural engineering	Public Sector auditing	Healthcare (cardiology)	Healthcare (oncology)	Healthcare (neurodevelopment)	Cybersecurity
Primary objective	Improve fabric defect detection via enhanced super-resolution	Accurate hysteresis modeling using interpretable networks	Interpret environmental data for sustainability decisions	Secure, real-time intrusion detection in IoV	Compensate encoder error for precision measurement	Predict EDSC compression capacity using ML	Review AI’s role in auditing, especially outside finance	Early cardiac disease detection using multi-modal data	Gene-based classification of breast cancer	Efficient ADHD detection using EEG	Detect zero-day exploits with high accuracy
Techniques used	Real-ESRGAN, Poisson Noise Simulation, Local Blur Loss	Dahl Model, Layer Recurrent Networks	ALBEF Transformer, VQA	DAGSNet, GAT, ViT, CMSO, SMPC, AES-256	VMD, DBN, PSO	ANN, SVR, RF, GEP, AdaBoost	Bibliometric Analysis, Case Studies	Federated Learning, ResNet50, Feature Fusion	Seagull Optimization Algorithm, Random Forest	NeuroDCT-ICA, RFO, ADHD-AttentionNet	AWPA, GMCO, MATA, AHEDNet
Data types used	Low-res fabric images	Actuator displacement/time data	Environmental images and text	CAN & CIC-IDS2017 datasets	Encoder readings + ambient temperature	Column geometry & load data	Audit logs, public data	ECG, echo, lifestyle & clinical records	Gene expression data	EEG signals	Network traffic
Preprocessing methods	Noise modeling, edge-aware learning	Time-series normalization	Vision-language alignment	Wavelet Transform, Encryption, Autoencoders	VMD for signal decomposition	Feature normalization	Data aggregation, cleaning	Median filtering, ICA, K-means	N/A	NeuroDCT-ICA	AWPA
Feature selection	Implicit via attention & discriminative loss	Physically derived parameters	Not required	CMSO Algorithm	PSO-optimized DBN	AdaBoost built-in	Case abstraction	Not emphasized	Seagull Optimization	RhinoFish Optimization	GMCO
Feature extraction	Fourier + Statistical + Residual maps	DahlNN + Recurrent dynamics	ALBEF cross-modal transformer	DAGSNet, GAT, ViT layers	DBN hierarchical learning	GEP-based encoding	N/A (review paper)	ResNet50, Dietary Patterns	Selected gene subsets	Decomposed EEG bands	MATA
Classifier/model	Real-ESRGAN + Custom Loss	Dahl-LRN Hybrid Model	ALBEF VQA	DAGSNet + CMSO + ViT	PSO-DBN	AdaBoost	N/A	SGD-optimized DNN	Random Forest	ADHD-AttentionNet	AHEDNet
Performance metrics	mAP↑, Recall↑, Precision↑ (vs baseline)	97.3% error reduction; 70% fluctuation reduction	Qualitative VQA output	Accuracy > 98%; fast encryption	Error reduced from 0.22° to 0.0025°	R^2^ = 0.996; MAPE = 0.013	Review – No metrics	Accuracy: 97.76–99.12%	Accuracy up to 99.01%	Accuracy: 98.52%, F1: 98.26%	Accuracy: 0.9919, Precision: 0.9968
Key innovation	Self-residual blur loss for local enhancement	Physically meaningful NN structure	VQA for sustainability	Hybrid encryption + ensemble deep model	Combined VMD and DBN with PSO	GUI-enabled fast prediction	Defines intelligent audit frameworks	Federated multimodal architecture	First SGA use for gene selection	Custom hybrid attention network	Multi-stage anomaly detection pipeline
Scalability	High – Data synthesis supports model expansion	High – Lightweight, interpretable	High – Generalizable framework	High – Secure and distributed	High – Temperature-aware model	High – GUI aids engineers	High – Framework adaptable	High – Decentralized FL model	High – Reduced gene input	High – EEG compatible	High – Generalizable to new threats
Real-time applicability	Yes—supports real-time defect inspection	Yes—Low computational cost	Moderate—Policy recommendation	Yes—Secure, fast detection	Yes—Fast prediction	Yes—Real-time design aid	Moderate—Depends on implementation	Yes—Clinical integration enabled	Yes—Real-time diagnosis	Yes—EEG-enabled screening	Yes—Real-time anomaly monitoring
Domain relevance	Strong—Textile quality inspection	Strong—High-precision robotics	Strong—Environmental decision-making	Strong—Vehicle cybersecurity	Strong—Robotic control systems	Strong—Civil design applications	Strong—Public sector efficiency	Strong—Clinical cardiology	Strong—Early cancer diagnosis	Strong—ADHD diagnostics	Strong—Cyber threat mitigation
Future scope	Extend to other materials, real-time integration	Apply to other actuators and sensors	Integration with climate data and AI agents	Edge AI and V2X integration	Apply to different encoders/motors	Extend to other column designs	Develop standard audit data models	More multimodal medical data, attack types	Extend to multi-cancer models	Include behavioral and clinical signals	Broaden detection to more attack types

The work evaluated three advanced meta-heuristic algorithms—GWO, SFS, and JADE—for optimizing the weight of a ten-story steel frame while satisfying AISC-LRFD constraints. GWO mimicked wolf hierarchy, SFS was inspired by fractal growth, and JADE functioned as a self-adaptive differential evolution variant. All three algorithms effectively met the design objectives^32^. The MOBBO algorithm, inspired by the foraging behavior of brown bears, was tested on five benchmark truss structures and achieved superior Pareto front convergence and diversity metrics. It ranked highest in HV and IGD, confirming its robustness, though it remained sensitive to initial conditions. Future directions included hybridization and real-world applications.

^33^. The MOCS2arc algorithm, which enhanced the traditional MOCS with a dual-archive strategy, was applied to eight truss structures and six ZDT functions. It consistently outperformed benchmark algorithms in minimizing mass and compliance. Despite minor limitations, MOCS2arc demonstrated strong adaptability and showed potential for hybrid optimization in complex engineering problems^34^.

## Proposed system

The proposed system addresses two critical challenges: reducing carbon emissions and enhancing the efficiency of global supply chains. By employing a combination of predictive modeling and optimization techniques, the system uses CDP report data to drive sustainable decision-making.

**Fig. 1:**
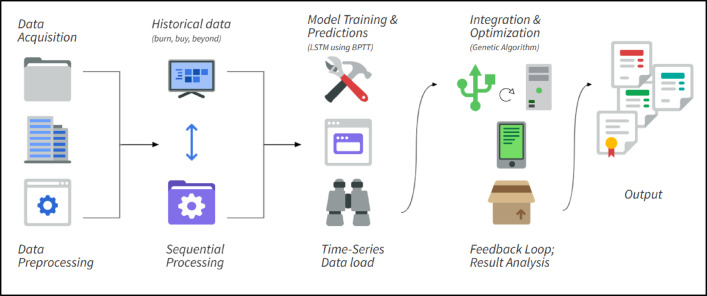
Proposed system framework.

As shown in Fig. 1, the framework combines both optimization and machine learning methodologies. The process begins with data collection and preprocessing, followed by time-series forecasting using LSTM networks trained via Backpropagation Through Time (BPTT).

GA is applied to optimize supply chain configurations by minimizing emissions while maintaining operational requirements. The iterative evolution of the GA helps refine emission reduction strategies based on LSTM predictions, ensuring a dynamic and adaptive decision-making process. The framework consists of the following four key stages:

1. Data Acquisition and Preprocessing: The first stage involves extracting operational parameters and emissions data from CDP reports, followed by normalization and organization for computational use.
2. GA Optimization: GA is used to explore the best supply chain configurations, aiming to minimize emissions while fulfilling operational demands.
3. LSTM-Based Emission Forecasting: Historical data is used to forecast future emission levels, which informs long-term sustainability planning.
4. GA-LSTM Integration: GA refines sustainability strategies based on the predictions generated by the LSTM network, ensuring continuous adaptation and optimization.

Together, these components form a robust and flexible system capable of meeting the evolving demands of supply chain sustainability.

**Fig. 2:**
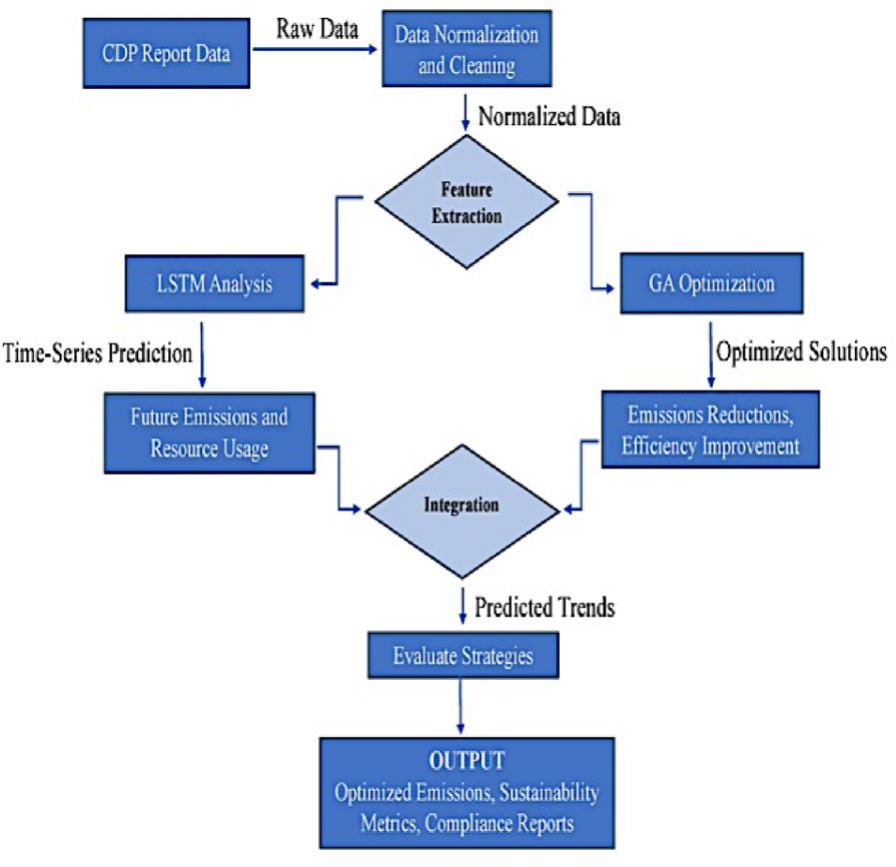
Process flow of the CDP.

Figure 2 explores the complete flow of the system, starting with CDP Report data extraction and concluding at the optimized output.

### Carbon disclosure project report

A global program called the CDP gathers and disseminates environmental data from cities and businesses with an emphasis on climate-related hazards, water use, and carbon emissions. Scope 1 (direct emissions from owned or controlled sources), Scope 2 (indirect emissions from purchased electricity, steam, heating, and cooling), and Scope 3 (indirect emissions from the entire value chain, including supply chain and product use) are the three emission scopes for which it encourages organisations to disclose information. To help stakeholders evaluate environmental consequences, monitor performance, and spot areas for improvement, CDP reports now include energy use, climate governance, and sustainability plans.

### Data acquisition and preprocessing

Extracting structured and unstructured data from CDP reports is the first stage of the process. In addition to other crucial sustainability indicators like energy, water, and waste management, these reports offer thorough information on organisational emissions across many scopes, which is necessary for well-informed decision-making. The CDP reports are parsed to arrive at the following critical information.

Direct Emissions: Fuel burning, industrial operations, and business cars are examples of owned or controlled sources of direct Scope 1 emissions.

Indirect Emissions: Indirect emissions from the production of steam, energy that is purchased, and heating and cooling that the business uses.

Semi-direct Emissions: Indirect emissions that happen throughout the supply chain, such as waste disposal, vendor operations, transportation, and emissions from the lifespan of a product.

To ensure interoperability with computational tools for additional optimisation, prediction, and sustainability research, these data are parsed, cleaned, and transformed into machine-readable forms.

### GA optimization

Natural selection serves as the inspiration for the GA, a population-based metaheuristic optimisation method. This study uses data from CDP reports to optimise supply chain emissions and operational efficiency using GA. This paradigm makes use of the organised and sequential metrics revealed in CDP reports, using Scope emissions (1,2,3), mobility modes, and energy use, in contrast to conventional methods that rely on IoT sensor data. Using data from the CDP, the GA is used to optimise business sustainability initiatives. By storing important variables including Scope 1, Scope 2, and Scope 3 emissions, the use of renewable energy, and the effectiveness of transportation into chromosomes, GA dynamically improves emissions reduction strategies in contrast to static optimisation techniques. In order to assure optimal solutions, the fitness function assesses plans based on energy efficiency, emissions reduction, and regulatory compliance, with penalties for non-compliance.

GA progressively improves sustainability metrics through mutation, multi-point crossover, and tournament-based selection. This method is scalable and economical as it allows organisations to model various sustainability situations without using real-time sensor data. By combining GA with LSTM forecasting, predictive skills are further improved, enabling businesses to foresee the long-term effects of their sustainability activities and coordinate with global climate commitments.

Every chromosome in the suggested GA framework is a possible way to optimise emissions and operational parameters that are obtained from CDP reports.

A vector is used to encode the chromosome as show in Eq. (1):

1 $$Chromosome:\left[ {E_{scope1} , E_{scope2} , E_{scope3} , T, R} \right]$$

To ensure uniformity and comparability across datasets, emissions are normalized using the formula given in Eq. (2).

2 $$E_{normalized} = \frac{{E_{actual} }}{{E_{max} }}$$

By striking a balance between three goals—reducing overall emissions, increasing operational effectiveness, and guaranteeing regulatory compliance—the fitness function assesses the quality of each chromosome. The fitness function that has been suggested in Eq. (3):

3 $$F\left( x \right) = w_{1} \cdot \frac{1}{{E_{total} }} + w_{2} \cdot \eta - w_{3} \cdot C_{penalty}$$

where, ${E}_{total}$ = ${E}_{scope1}+ {E}_{scope2}+ {E}_{scope3}$. It represents total emissions aggregated across all scopes, $\eta = \frac{Optimized output}{Baseline Input}$ indicates operational efficiency ratio derived from CDP-reported resource utilization, ${C}_{penalty}$ denotes the penalty term for deviations from regulatory or sustainability benchmarks and ${w}_{1}, {w}_{2}, {w}_{3}$ are the weight factors that prioritize emissions reduction, efficiency, and compliance, respectively.

Selection, crossover, and mutation are the three main genetic operators used by the GA, which iteratively develop the population in search of the best answer. Parent chromosomes are chosen via selection according to their fitness values. Better solutions are indicated by higher fitness levels. Fitter chromosomes have a greater chance of reproducing if strategies like tournament selection or roulette wheel are used. By fusing two parent chromosomes to create offspring, crossover makes it easier to investigate novel solutions. It employs a single-point crossover, which is described in Eq. (4) as:

4 $$Offspring = \alpha \cdot Parent_{1} + \left( {1 - \alpha } \right) \cdot Parent_{2}$$

where, $\alpha$ represents the crossover rate (0 ≤ α ≤ 1) and ${Parent}_{1}, {Parent}_{2}$ are parent chromosomes.

Mutations prevent premature convergence to local optima by introducing random mutations to the chromosomes of children. Mutation mimics changes in operational choices, such switching to renewable energy sources or different means of transportation, for emissions parameters, as depicted in Eq. (5):

5 $$E_{mutated} = E_{gene} \cdot \left( {1 + \delta } \right)$$

where, ${E}_{gene}$ denotes original emission value and $\delta$ :is the random perturbation factor (− 0.1 ≤ $\delta$  ≤ 0.1).

### Long short-term memory for predictive modelling

RNNs of the LSTM network type are made to capture long-term relationships to represent and analyse sequential data. Because LSTMs excel at time-series forecasting, they are perfect for examining past emissions data and resource utilisation trends included in CDP databases. LSTMs forget, input, and output gates allow them to learn complex temporal correlations without experiencing vanishing gradient issues.

Based on past CDP data, predictive modelling using LSTM networks is used to project corporate carbon emissions. LSTM is ideally suited for sustainability forecasting because, in contrast to conventional regression models, it efficiently captures long-term relationships and patterns in time-series data. Accurate forecasts of future environmental performance are made possible by LSTM, which examines historical patterns in energy consumption, emissions, and the adoption of renewable energy sources in Scope 1, Scope 2, and Scope 3.

The model learns temporal correlations to predict future trends by using previous emissions data as training data. By proactively modifying their sustainability policies, organisations may maximise their efforts to reduce emissions thanks to this predictive capabilities. The model dynamically improves sustainability plans by combining LSTM and the GA, guaranteeing that business environmental policies are data-driven, flexible, and in line with global sustainability targets.

Time-series data, including changes in emissions from year to year, patterns of energy use, and operational objectives, are frequently included in CDP reports. These reports often provide detailed insights into how companies are adapting their strategies to meet sustainability goals, reduce carbon footprints, and align with global climate targets over time. The input sequence would look as it does in Eq. (6):

6 $$X = \left\{ {x_{1} , x_{2} , x_{3} , \ldots \ldots x_{T} } \right\}$$

where, X: Input sequence containing CDP metrics, ${x}_{t}=$[${E}_{scope1,t}, {E}_{scope2.t}, {E}_{scope3,t}, {R}_{t}, {T}_{t}]$: Feature vector at time t, including: ${E}_{scope1,t}, {E}_{scope2.t}, {E}_{scope3,t}$ : Scope 1, 2, and 3 emissions at time t, ${R}_{t}$: Renewable energy usage ratio and ${T}_{t}$: Transportation mode indicator.

The model is trained by minimizing the MSE loss function as displayed in Eq. (7):

7 $$Loss = \frac{1}{N} + \mathop \sum \limits_{i = 1}^{N} \left( {\hat{y} - y_{i} } \right)^{2}$$

where, $\widehat{y}$ : Predicted value for the *i*—th sample, ${y}_{i}$ : Actual value for the *i*—th sample and $N$ : Number of training samples.

The training process involves using the LSTM model, which is trained on historical CDP data. The training employs the BPTT algorithm to effectively handle sequential data dependencies and ensure the model learns from long-term relationships. For optimizing the model parameters, the Adam optimizer is utilized. This optimizer combines the benefits of momentum and adaptive learning rates, ensuring faster convergence and improved model accuracy. This is depicted in Eq. (8):

8 $$\theta \leftarrow \theta - \eta \cdot \nabla Loss$$

where,$\theta$: Trainable parameters, $\eta$: Learning rate.

### GA-LSTM integration

Based on data from the CDP, the combination of GA and LSTM networks offers a unique, hybrid method for handling the challenges of supply chain emissions control. The combined approach makes use of both algorithms’ advantages: LSTM’s ability to identify sequential patterns in time-series data and GA’s ability to optimize multi-objective problems. A strong system for lowering emissions, increasing operational effectiveness, and adhering to sustainability objectives is the outcome of this synergy.

Time-series emissions data from CDP reports are modelled and predicted using the LSTM network. It anticipates future metrics including Scope 1, Scope 2, and Scope 3 emissions, as well as energy consumption and transportation patterns, and it records temporal dependencies. Future sustainability scenarios may be incorporated into the optimisation process by using the GA’s input, which is the predictions produced by the LSTM network. This integrated approach enables organizations to identify optimal strategies.

The output of this LSTM is represented as shown in Eq. (9):

9 $$\widehat{{y_{T} }} = W_{y} \cdot h_{T} + b_{y}$$

where, $\widehat{{y}_{T}}$ represents predicted emissions or sustainability metric at time T + 1, ${h}_{T}$ denotes Hidden state of the LSTM at the final time step T and ${W}_{y}, {b}_{y}$ is Trainable output weight matrix and bias.

The GA optimises the LSTM’s forecasts of future sustainability indicators to reduce emissions and increase operational effectiveness. In order to carry out this optimisation, the GA compares potential solutions (chromosomes) to a fitness function as shown in Eq. (3). The integration of GA and LSTM involves a feedback loop where, The LSTM predicts future emissions based on historic data, Eq. (10).

The GA uses LSTM predictions to identify the best optimization strategies. This is depicted in Eq. (11).

10 $$\widehat{{y_{T} }} = LSTM\left( X \right)$$

11 $${\text{F}}\left( {\text{X}} \right) \, = {\text{ Fitness}}\,{\text{ Function}}\,{\text{ Evaluation}}$$

The integration guarantees that optimization decisions are led by accurate projections of sustainability measures. The LSTM’s capacity to predict future trends complements the GA’s capability to discover optimum solutions, resulting I an equation depicted in Eq. (12).

12 $${\Delta E} = { }\frac{{E_{baseline} - E_{optimized} }}{{E_{baseline} }} \times 100$$

where, $\Delta E$ is the percentage reduction in emissions after optimization, ${E}_{baseline}$ is the predicted emissions without optimization and ${E}_{optimized}\text{ is the}$ Emissions after applying GA-optimized solutions.

The uniqueness of this hybrid technique is its capacity to:

Combine sequential pattern recognition (with LSTM) and multi-objective optimisation (using GA).

- Use CDP-reported metrics as the only input, removing the requirement for real-time IoT data.
- Provide practical insights into emissions reduction and sustainability planning via scenario-based forecasting and optimisation.
- This technique uses predictive modelling and optimization to align sustainability goals with operational decisions.

## Results and discussion

The experimental results validate the effectiveness of the proposed GA-LSTM hybrid framework for optimizing supply chain sustainability using CDP-reported data. The approach combines the predictive capabilities of LSTM networks with the optimization strength of GA to reduce carbon emissions, improve operational efficiency, and support informed decision-making.

### Emissions reduction and operational impact

**Fig. 3:**
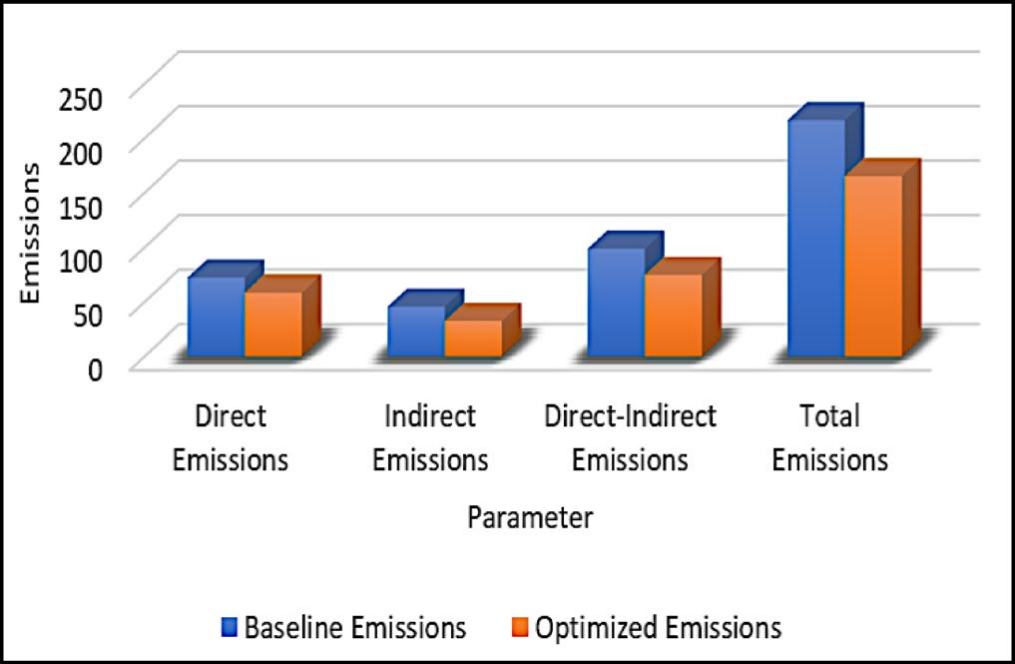
Comparisons of emissions before and after optimization.

The system demonstrated a notable reduction in emissions across three scopes: Direct, Indirect, and Direct–Indirect. Table 2 summarizes the emissions before and after optimization. The proposed method achieves a 23.67% reduction in overall emissions, demonstrating its effectiveness in optimizing carbon footprint management. Figure 3 visually highlights the substantial reduction in emissions post-optimization. The highest reduction was observed in Indirect emissions, emphasizing the effectiveness of integrating clean energy and efficient transport strategies.

**Table 2:** Comparison of emissions before and after optimization.

Parameter	Baseline emissions	Optimized emissions	Reduction (%)
Direct emissions	72.1	58.4	18.99
Indirect emissions	45.6	32.3	29.17
Direct–indirect emissions	98.7	74.5	24.52
Total emissions	216.4	165.2	23.67

### Convergence behavior of the genetic algorithm

**Fig. 4:**
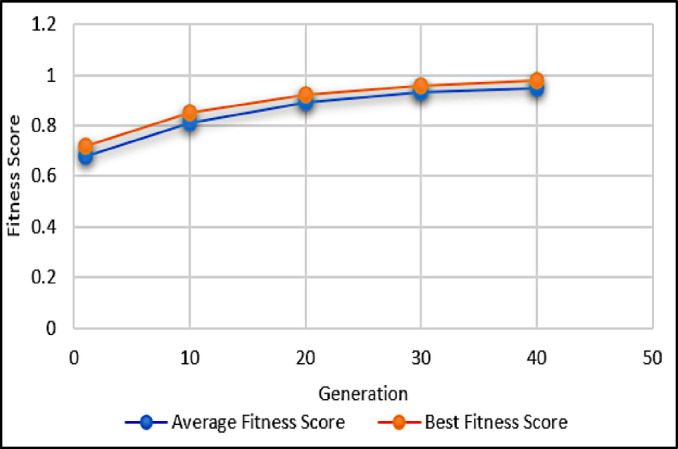
Fitness score vs. generations.

Table 3 demonstrates the convergence of GA over multiple generations. A consistent increase in both average and best fitness scores indicates that the optimization process effectively improves sustainability objectives. Figure 4 supports this trend with a steady fitness score.

**Table 3:** Fitness score convergence.

Generation	Average fitness score	Best fitness score	Improvement (%)
1	0.68	0.72	–
10	0.81	0.85	18.06
20	0.89	0.92	8.24
30	0.93	0.96	4.35
40	0.95	0.98	2.08

### Renewable energy and emission correlation

**Fig. 5:**
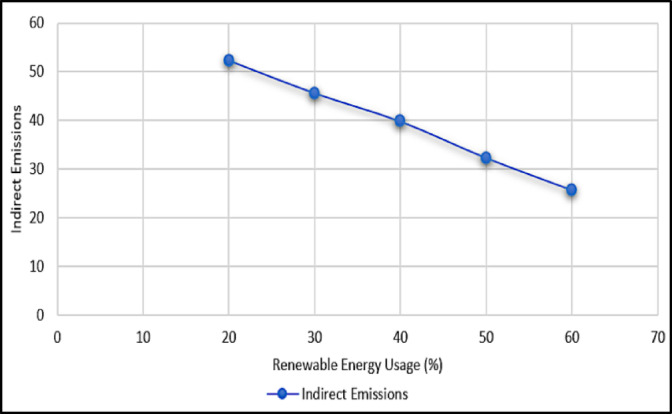
Emission vs. renewable energy usage.

Table 4 reveal a strong inverse correlation between renewable energy adoption and Indirect emissions, reinforcing the strategic value of increasing clean energy usage. This highlights the environmental benefits of switching to cleaner energy sources. Figure 5 illustrates this relationship by visually displaying how increased renewable energy use leads to large reductions in emissions.

**Table 4:** Renewable energy adoption and emission reduction.

Renewable energy usage (%)	Indirect emissions (%)	Indirect emission reduction (%)
20	52.3	0
30	45.6	12.81
40	39.8	23.86
50	32.3	38.27
60	25.7	50.86

### Transport optimization for sustainability

**Fig. 6:**
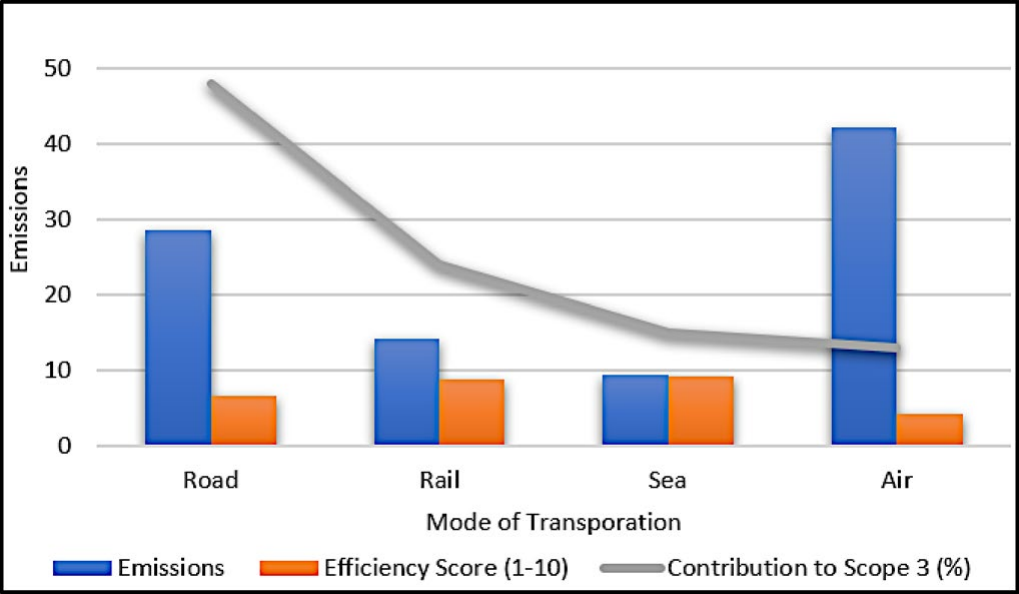
Transportation emissions vs. efficiency.

Table 5 compares transportation modes based on emissions and efficiency scores. As visualized in Fig. 6, modes like sea and rail provide a better sustainability-to-efficiency ratio than road and air. Table 4 and Fig. 6 highlight the possibility for transportation plan changes to lower environmental footprints while maintaining performance.

**Table 5:** Transportation mode efficiency analysis.

Mode	Emissions	Efficiency score (1–10)	Contribution to direct–indirect (%)
Road	28.5	6.5	48
Rail	14.2	8.7	24
Sea	9.3	9.1	15
Air	42.1	4.2	13

### Feature importance in LSTM predictions

Table 6 shows the importance of input features to LSTM predictions. It demonstrates that Direct–Indirect emissions, which represent indirect supply chain emissions, are the largest contributors, followed by Direct emissions. Furthermore, incorporating renewable energy usage and transportation modes is critical to improving the accuracy of emissions estimates.

**Table 6:** Feature importance for LSTM predictions.

Feature	Importance Score (%)	Contribution explanation
Direct emissions	27.3	Direct operational emissions from the organization
Direct–indirect emissions	31.5	Indirect supply chain emissions largest contributor
Renewable energy usage	18.7	Contribution of renewables to operational energy mix
Transportation mode	12.4	Efficiency and sustainability of logistics
Energy usage	10.1	Resource consumption patterns

### Multi-objective optimization results

Table 7 shows the trade-offs made during the optimization process, which involved balancing emission reductions, operating efficiency, and compliance. The technology achieves reduction in emissions, increase in operational efficiency, and eliminates compliance penalties.

**Table 7:** Multi-objective trade-offs.

Objective	Baseline value	Optimized value	Trade-off (%)
Total emissions	216.4	165.2	− 23.67
Operational efficiency	0.82	0.91	+ 10.98
Compliance penalty	5.3	0	− 100

**Fig. 7:**
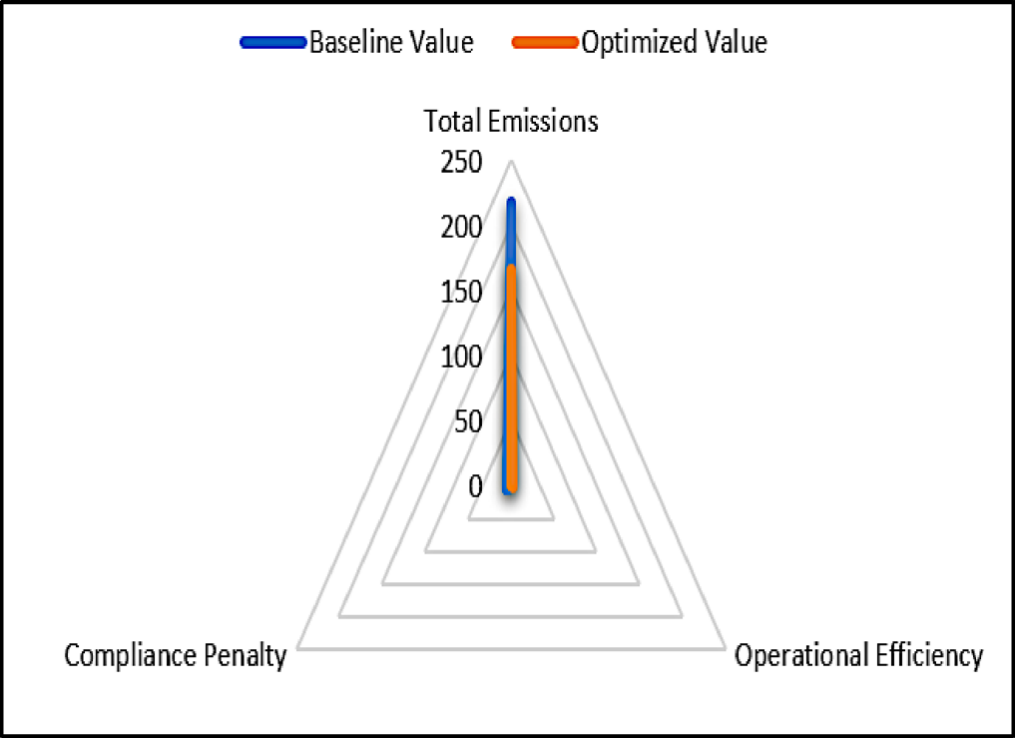
Multi-objective optimization.

Figure 7 employs a radar graph to contrast the baseline and optimum values for emissions, operational efficiency, and compliance. It depicts the multi-objective optimization process, demonstrating how the system simultaneously decreases emissions, increases efficiency, and assures compliance. The findings address the study’s primary objectives by proving that hybrid AI frameworks can analyse sequential data, maximize sustainability measures, and provide actionable insights for informed decision-making.

The important findings are summarized as follows: For greater sustainability, CDP-reported data, including energy consumption, emissions, and transportation statistics, were employed instead of real-time IoT sensors. This approach provides a widely accessible and cost-effective sustainability analysis option. Significant decreases in Scope emissions were achieved by the hybrid framework, which showed visible benefits due to the Genetic Algorithm’s multiple goals of metrics predicted by the LSTM model. Process optimization increased operating efficiency by a measurable 10% while reducing environmental impact. Efficient resource management was necessary to meet sustainability targets. Through resource usage optimization based on expected demand and availability, energy consumption was significantly reduced, waste was minimized, and production outputs were in line with capacity.

The framework’s effectiveness in meeting sustainability goals was confirmed by eliminating compliance penalties, ensuring adherence to benchmarks and regulations. It combined LSTM networks for accurate emissions and resource trend predictions with Genetic Algorithms to optimize emission reductions and efficiency improvements. This approach offered actionable insights that align with both environmental goals and business objectives, addressing complex environmental and operational challenges. The study’s findings indicate that combining GA with LSTM could lead to revolutionary advances in supply chain management. Reduced emissions and improved efficiency are two advantages of the hybrid approach, which only uses CDP-reported data. These findings lay the basis for future research aimed at improving AI-driven techniques for even bigger effects, whilst supporting the overall objective of encouraging sustainable habits. The findings demonstrate the feasibility and scalability of this strategy, as well as significant insights into how AI might be used to operational and environmental objectives.

### Comparative performance analysis with existing emission reduction methods

**Fig. 8:**
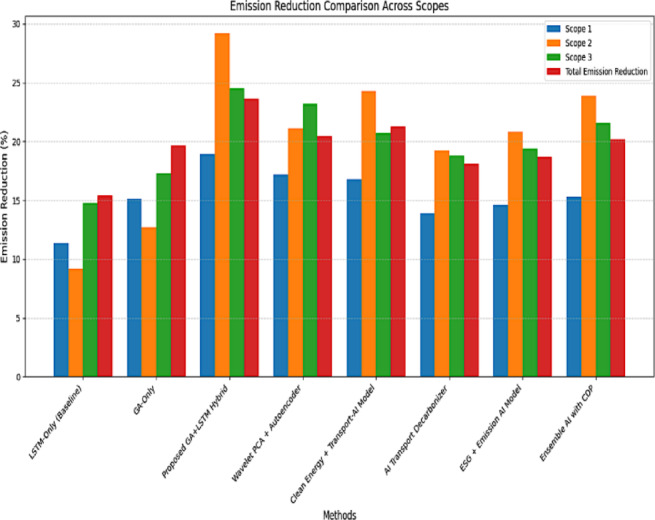
Comparison of emission reduction.

The results presented in Fig. 8 clearly demonstrate the superior performance of the proposed GA + LSTM hybrid model in reducing greenhouse gas emissions across all three emission scopes. Achieving a total emissions reduction of 23.67%, it significantly outperforms traditional LSTM-only models and other recent AI-based approaches. Notably, the model achieves a 29.2% reduction in Scope 2 emissions, highlighting its effectiveness in optimizing energy consumption and improving sustainability in operations that rely heavily on purchased electricity. Unlike other methods that may perform well in isolated scopes, the GA + LSTM model ensures balanced and consistent improvements across Scope 1, Scope 2, and Scope 3, indicating its robustness and adaptability in diverse emission scenarios. This hybrid strategy not only enhances forecasting accuracy through LSTM but also improves model inputs and convergence using GA, resulting in more actionable and impactful emission control. Therefore, the proposed system stands out as a highly effective tool for comprehensive decarbonization in smart transportation and energy-intensive domains.

### Strengths, limitations, and broader implications

#### Key strengths

The GA-LSTM hybrid framework showcases notable advantages, particularly in achieving substantial emission reductions (23.67%) across all scopes using only publicly available CDP-reported data. This eliminates reliance on expensive real-time IoT infrastructure, making the solution highly accessible. The multi-objective optimization capability enables simultaneous gains in emissions control, operational efficiency (10.98%), and full regulatory compliance. Moreover, the framework’s flexibility allows for its application across various industry domains by adjusting sector-specific input parameters.

#### Identified Limitations

Several limitations must be acknowledged. First, the absence of real-time sensor data limits the model’s adaptability to sudden operational disruptions. Second, the framework’s accuracy is inherently tied to the quality and completeness of the historical CDP data used. Third, while basic feature importance analysis was performed, more advanced interpretability techniques such as SHAP or LIME were not explored. Finally, the model’s validation is currently limited to simulations, and real-world deployment remains to be tested.

#### Practical significance

This framework offers a low-cost yet effective pathway for organizations especially SMEs to engage in sustainability optimization. By leveraging publicly reported data and lightweight AI models, it delivers strategic insights into emissions trends, renewable energy adoption, and transportation efficiency. The approach supports practical integration into corporate decision-making systems, including ESG monitoring and sustainability reporting platforms.

#### Broader AI contribution to sustainability

This research aligns with global efforts to employ AI for environmental sustainability. AI-driven models like the one proposed can support carbon footprint reduction, resource optimization, and compliance with sustainability standards. The GA-LSTM framework exemplifies how accessible AI tools can empower organizations to align operational goals with global climate commitments, contributing meaningfully to long-term sustainability objectives such as the UN SDGs.

## Conclusion and future works

As the global focus on sustainability intensifies, there is an increasing need for innovative solutions that balance environmental responsibility with operational efficiency. This study presents a novel AI-driven framework that integrates predictive modeling and multi-objective optimization for enhancing sustainability in supply chain management. By combining the strengths of GA and LSTM networks, the proposed framework provides a dynamic, data-driven approach to reducing carbon emissions, improving operational efficiency, and ensuring regulatory compliance. This hybrid approach goes beyond traditional rule-based models by integrating predictive modeling with multi-objective optimization to balance emissions reduction, cost-efficiency, and operational performance. By utilizing structured emissions data from CDP reports, the framework optimizes emissions across Scope 1, Scope 2, and Scope 3, leading to a 23.67% reduction in carbon emissions and a 10.98% improvement in operational efficiency. It also guarantees regulatory adherence with a 100% elimination of compliance penalties. The integration of LSTM networks for accurate emission forecasts and GA for optimizing operational decisions provides a robust mechanism for driving meaningful sustainability improvements. Despite its significant contributions, the study acknowledges certain limitations. The quality and consistency of CDP-reported data play a crucial role in the framework’s effectiveness, with incomplete or inconsistent data leading to suboptimal results. Additionally, the computational demand of LSTM and GA training phases, although lower than sensor-based alternatives, still requires substantial resources. Future improvements could focus on enhancing data collection methods and optimizing algorithmic designs to reduce computational costs.

Looking forward, the integration of real-time IoT data can enhance model adaptability, while exploring Reinforcement Learning (RL) for continuous optimization could make the framework more dynamic. The application of Explainable AI (XAI) techniques like SHAP or LIME could improve model interpretability, building stakeholder trust. Future research may also explore scalability across diverse industries to test the framework’s versatility. The incorporation of emerging technologies, such as blockchain, could further enhance transparency and data integrity. By addressing these future challenges and continuously evolving the model, this research paves the way for scalable, AI-driven solutions that align business objectives with sustainability goals, fostering a greener and more sustainable future.

## Data Availability

Data is provided within the manuscript.
